# Therapeutic targeting of alternative pathway and C5 but not C5a protects from disease development in a preclinical model of autoimmune blistering dermatosis

**DOI:** 10.3389/fimmu.2025.1560468

**Published:** 2025-04-30

**Authors:** Björn Laffer, Mareike Ohms, Samyr Kenno, Ping Tsui, Elvira Ehlers-Jeske, Wenru Song, Wen-Chao Song, Jörg Köhl

**Affiliations:** ^1^ Institute for Systemic Inflammation Research, University of Lübeck, Lübeck, Germany; ^2^ Kira Pharmaceuticals, Research and Development, Cambridge, MA, United States; ^3^ Department of Systems Pharmacology and Translational Therapeutics, The University of Pennsylvania, Philadelphia, PA, United States

**Keywords:** EBA, complement, C5a, alternative pathway, bullous pemphigoid

## Abstract

**Introduction:**

Epidermolysis Bullosa Acquisita (EBA) is an autoimmune blistering dermatosis characterized by autoantibodies (AAbs) against type VII collagen (COL7) located at the dermal epidermal junction (DEJ). Local complement activation drives C5a generation associated with neutrophil recruitment and activation resulting in skin lesions and inflammation. Here we tested the impact of C5a/C5adesArg, C5 or combined C5 and alternative pathway (AP) targeting on disease development and skin inflammation in a preclinical mouse model mimicking the effector phase of EBA.

**Methods:**

C57BL/6 mice were treated subcutaneously with purified rabbit anti-mouse-COL7 IgG in the presence of IgG1 mAbs directed against murine C5a/C5adesArg (M031), C5 (mBB5.1), a bifunctional protein comprising mBB5.1 fused to an active fragment of the AP inhibitor factor H (M014) or an IgG1 isotype control mAb. Formation of skin lesions was evaluated 12 days every other day. On day 12, DEJ separation, IgG AAb and C3b deposition and neutrophil infiltration was assessed.

**Results:**

Isotype IgG1-treated mice developed first skin lesions on day 4 peaking on day 12. Prophylactic treatment with either M031 or M014 markedly reduced the development of skin lesions, the dermal/epidermal separation and neutrophil recruitment. Surprisingly, C5 or combined AP/C5 inhibition by M014 but not C5a/C5adesArg-targeting by M031 reduced the development of skin lesions and dermal/epidermal separation in the setting of therapeutic treatment. IgG and C3b deposition was not affected by either treatment. Importantly, direct comparison of isolated C5 targeting by mBB5.1 vs. combined AP/C5 inhibition by M014 revealed that M014 reduced the development of skin lesions earlier and more pronounced than mBB5.1.

**Discussion:**

Our findings identify combined C5/AP targeting as a novel therapeutic option for autoimmune blistering dermatoses.

## Introduction

Autoimmune blistering dermatoses (AIBD) comprise a group of diseases including bullous pemphigoid (BP), Epidermolysis bullosa acquisita (EBA), mucus membrane pemphigoid and pemphigoid gestations, among others, which manifest in the skin and mucous membranes (1). The disease develops in response to tissue-bound autoantibodies (AAb) targeting different structural proteins in the skin. In case of EBA, the auto-antigen is the non-collagenous domain of type VII collagen (COL7). This protein is needed for the attachment of the epidermis to the underlying dermis, building the dermal-epidermal junction (DEJ). EBA is a heterogenous disease appearing clinically and histologically either as the mechanobullous classic form or the inflammatory subtype (1). Histologically, the inflammatory EBA subtype is characterized by dermal-epidermal separation (2, 3) and an increased abundance of neutrophils that release reactive oxygen species (ROS) and proteases at the basement membrane zone, which drive dermal-epidermal separation and the development of blisters (4).

The tissue-bound immune complexes comprising COL7-directed IgG AAbs activate the complement system, which serves as an important effector system promoting the inflammatory response in EBA. Mouse models that mimic the disease histologically, clinically, and immunologically have contributed substantially to the current state of knowledge on the role of the complement system in EBA pathogenesis (5). EBA can be induced passively by the transfer of rabbit anti-mouse-COL7 IgG or actively by immunization of mice with an immunodominant domain of the murine COL7 antigen (6). In an active EBA mouse model, diseased mice showed higher C3b skin deposition than control animals as well as an elevation of complement fixing IgG2a and IgG2b antibody subclasses (7). Furthermore, studies from Mihai et al. demonstrated that activation of the alternative pathway (AP) is required to induce EBA in a passive model since factor B-deficient mice and anti-factor B-treated mice showed delayed and decreased development of skin lesions, whereas deficiency in the classical, lectin or terminal pathway had no or only a minor impact (8, 9). Also, the C5-C5aR1/C5aR2 axis is engaged in the pathogenesis of EBA. C5-deficient mice or animals treated with an anti-C5 mAb showed a reduced clinical phenotype (9–11). Moreover, C5aR1-deficiency or pharmacological blockade of this receptor (9, 12) as well as C5aR2 deficiency (13, 14) resulted in disease protection in a passive EBA mouse model.

Thus, the available data suggest that pharmacological inhibition of the AP or downstream blockade of the C5/C5aR1 pathway could serve as novel therapeutic approach in EBA. To assess the contribution of C5 and AP targeting on EBA development, we generated a bifunctional mAb fusion protein M014, a modified version of the C5-specific mouse IgG1 mAb BB5.1 (mBB5.1) (15) fused to murine Factor H (FH) short consensus repeats (SCR)1-5. To compare the effect of combined AP and C5 targeting with that of C5 targeting alone we used mBB5.1 in the passive EBA model. Finally, we sought to determine the effect of targeting C5a and C5adesArg, the primary degradation product of C5a lacking the C-terminal arginine residue, which both bind to C5aR1 and C5aR2. C5adesArg emerges after removal of the C-terminal arginine residue from the C5a molecule by serum or tissue-derived carboxypeptidases (16) For this purpose, we treated mice with mAb M031, a chimeric mouse/rat IgG1 mAb specifically targeting murine C5a and C5adesArg. M014 and M031 were tested in a prophylactic and therapeutic treatment regimen. MAb mBB5.1 was tested only in the therapeutic approach.

## Methods

### Mice

C57BL/6J wild-type mice were bred and housed in a 12-hour light/12-hour dark cycle at the animal facility of the University of Lübeck. All experiments were approved by the Schleswig-Holstein state government (AZ 39 (71-10_21) and performed on 8- to 15-week-old age- and sex-matched mice by certified personnel.

### Antibody transfer-induced EBA model

Passive transfer studies followed published protocols (6, 10). Briefly, mice were treated with three sub-cutaneous (s.c.) injections of purified rabbit anti mouse-COL7 antibody (100 µg in 100 µl) on day 0 (into the neck), day 2 (into the right front leg) and day 4 (into the left hind leg) to induce skin lesions. For the prophylactic approach M031, M014 or the mouse anti-Hen Egg Lysozyme (HEL) IgG1 antibody (isotype control) was injected i.p. (50 mg/kg body weight) on days -1, 2, 5 and 8. For the therapeutic approach M031, M014, mBB5.1 or the isotype control IgG1 antibody was administered i.p. (50 mg/kg body weight) on days 5 and 8. The formation of skin lesions was evaluated using a scoring protocol on days 2, 4, 6, 8, 10 and 12 as described (17). Anesthesia was induced by i.p. injection of a mixture of ketamine (100 mg/kg body weight) and xylazine (7,5 mg/kg body weight). At the end of the experiment on day 12, mice were sacrificed, and skin biopsies were taken for immunohistochemical examination to determine neutrophil infiltration, tissue-bound IgG and complement C3b.

### Antibodies

For the antibody transfer-induced EBA model, we used rabbit antibodies directed against the von Willebrandt factor type A2 (vWFA2) domain of COL7 as described (11). For prophylactic and therapeutic treatment, M031, M014, modified anti-mouse C5 mAb mBB5.1 (15) and the anti-HEL mAb were produced and provided by Kira pharmaceuticals. M031 is a chimeric mouse/rat IgG1 mAb specifically targeting murine C5a and C5adesArg. M014 is a bifunctional mouse IgG1 mAb based on the structure of the mBB5.1 mAb (15) fused C-terminally in the heavy chain to murine FH SCR1-5, thereby blocking C5 cleavage by CP and AP pathway convertases and AP activation. The target molecules of the different antibodies used in the study are detailed in **Table 1**. Tissue-bound IgG AAb and C3b deposition was determined by direct IF microscopy of frozen sections in TissueTek (Sakura; Ref: 4583) using AF594-conjugated Donkey anti-rabbit IgG (H+L) (Jackson Immuno Research, No: 711-585-152, 3 μg/mL), or FITC-conjugated Goat IgG Fraction to mouse complement C3b (MP Biomedicals; No: 55500; 66 μg/mL). MPO^+^ or Ly6G^+^ cells were detected by direct IF microscopy of frozen ear sections in Tissue-Tek (Sakura, Ref4583) using FITC conjugated IgG antibodies directed against mouse MPO (Hycult, No: 23301M117-A) at a concentration of 20 μg/mL or AF594-conjugated rat anti-mouse Ly6G IgG (Biolegend, No: 127636, clone 1A8) at a concentration of 1 μg/mL. Cell nuclei was stained with DAPI (Life Technologies, CAT D3571, 5 μg/mL).

**Table 1:** Antibodies used in the study and their respective target molecules.

Antibodies	Target Structures			
	Hen egg lysozyme	C3b	C5	C5a/C5a desArg
IgG1 control	+	–	–	–
M014	–	+	+	–
M031	–	–	–	+
mBB5.1	–	–	+	–

### Immunohistochemical staining

Tissue-bound IgG AAbs and C3b deposition as well as MPO^+^ or Ly6G^+^ cells were identified by direct IF microscopy of frozen ear sections as described (18). In detail, cryosections on slides (SuperFrost Ultra Plus™ GOLD adhesion objective slides, 11976299, Epredia^™^) were fixed with acetone, washed with D-PBS and blocked with 1% Tween-20 in PBS + 1% BSA. For C3b/IgG staining, 10% goat serum and 10% donkey serum was added. The cryosections on slides were incubated for 60 min in a staining chamber (eBioscience Stain Tray) at room temperature (RT). After washing with D-PBS, cryosections on slides were incubated for 5 min at RT with DAPI in PBS. After washing with D-PBS a drop of Fluoroshield (Sigma Aldrich F6182) was added to the sections and covered with a cover slip. Sections were stored in a slide folder protected from light at 4°C.

For quantitative evaluation, we determined the area in µm^2^ and the mean fluorescence intensity (MFI) of rabbit IgG and C3b positive staining as well as MPO^+^ or Ly6G^+^ cells as described (18). Staining was evaluated using the Keyence BZ-X810 microscope with the BZ-X800 viewer and analyzer software (Basic Analysis Software and Advanced Observation Module). To quantify IgG AAb or C3b deposition and MPO^+^ or Ly6G^+^ cells, (100x magnification), we evaluated 24 pictures in overlay.

### Histopathology

Frozen ear sections were stained with the Kwik-Diff™ staining kit (Epredia™, No: 9990700) to evaluate the formation of subepidermal clefts at the dermal epidermal junction (DEJ). The staining was evaluated using the Keyence BZ-X810 microscope with the BZ-X800 viewer and analyzer software (Basic Analysis Software and Advanced Observation Module). To determine the percentage of dermal-epidermal separation, three different sections (100x magnification, 24 pictures in overlay) of one slide were evaluated and the average was calculated.

### Statistical analysis

For statistical analysis, the GraphPad PRISM 10 software was used. The data obtained were analyzed for normal distribution by the Kolmogorov-Smirnov test. Differences between two groups were assessed by unpaired t-test. Differences between three groups were determined by a one-way ANOVA with Dunnett’s posthoc or Holm-Šídák’s posthoc multiple-comparisons test. Differences were considered as significant at *p< 0.05, ** p<0.01, ***p <0.001, and ****p < 0.0001.

## Results

### Prophylactic treatment with M031 or M014 prevents mice from the development of skin lesions in passive EBA

**Figure 1:**
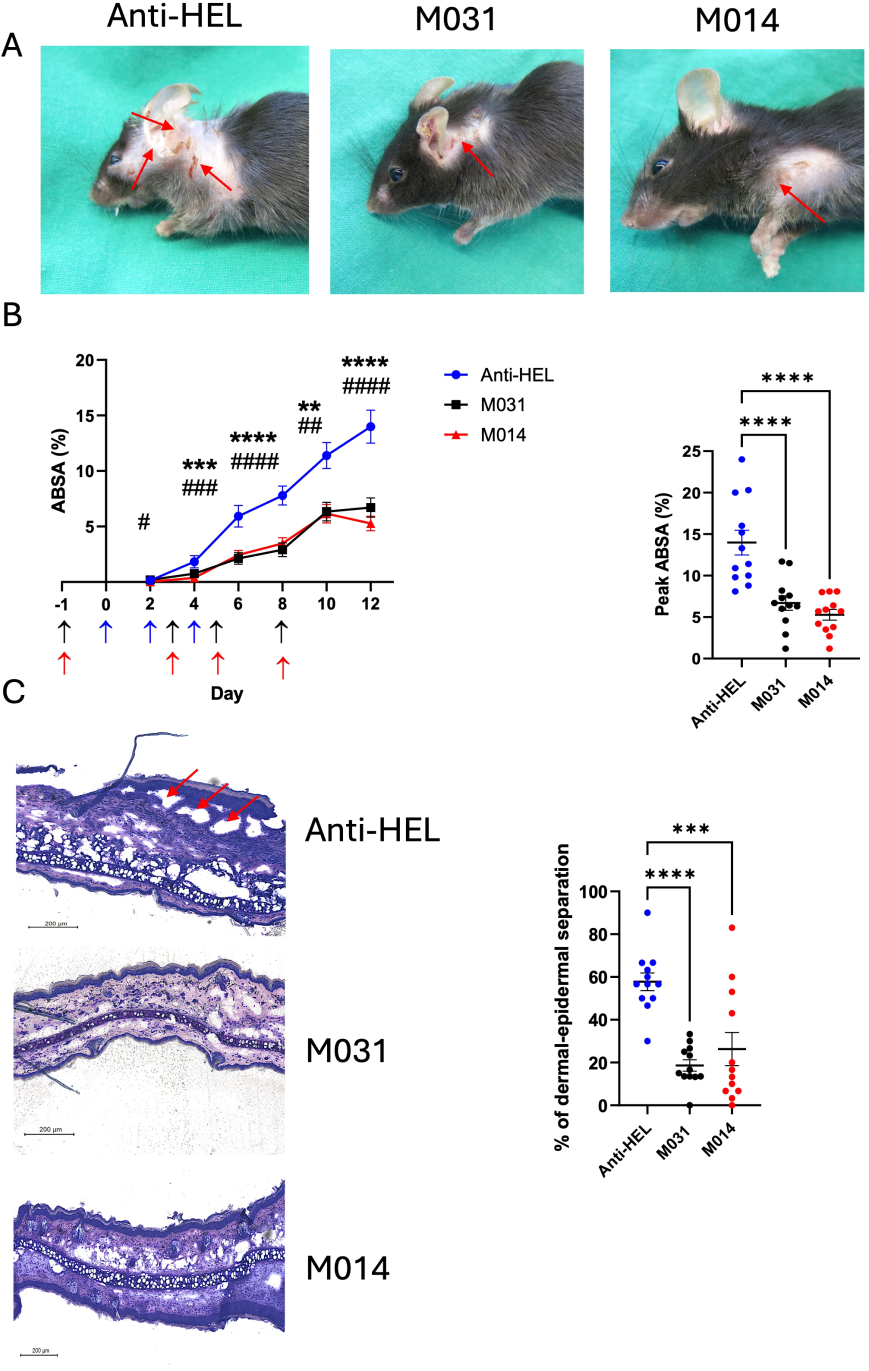
Prophylactic inhibition of C5a/C5adesArg by M031 or combined targeting of AP/C5 by M014 reduces the formation of skin lesions. **(A)** Representative picture of mice on day 12 after prophylactic treatment with anti-HEL control IgG1 mAb, M031 or M014 on days -1, 2, 5 and 8. Red arrows point toward skin lesions. **(B)** (Left panel) Cumulative disease scores benchmarked as percentage of the total body surface area affected by skin lesions (ABSA) of mice treated with anti-HEL mAb (blue), M031 (black) or M014 (red). Blue arrows show the time point when COL7-specific IgG AAbs were injected. Black (M031) or red (M014) arrows show the time points when complement inhibitors were administered. Results were pooled from 3 independent experiments. Statistical differences between groups were determined by One way ANOVA with Holm-Šídák’s posthoc multiple-comparisons test. **p < 0.01, ***p < 0.001. ****p < 0.0001 for M031-treated compared with HEL-IgG1 treated mice and ^#^p<0.05, ^##^ *p* < 0.01, ^###^ *p* < 0.001. ^####^ *p* < 0.0001 for M014-treated compared with HEL-IgG1-treated mice.; (right panel) peak value of ABSA assessed for each mouse. Scatter plots show the mean ± SEM (*n* = 12 mice per group). ****p<0.0001. **(C)** (Left panel) Histopathologic evaluation of dermal-epidermal separation. Shown are presentative pictures of skin sections from mice treated with anti-HEL IgG1, M031 or M014 on day 12. Red arrows indicate subepidermal clefts; (right panel) percentage of dermal-epidermal separation determined individually for each mouse treated with anti-HEL IgG1 (blue), M031 (black) or M014 (red). Results were pooled from 3 independent experiments. The scatter plots show the mean ± SEM (*n* = 12 mice per group). Statistical differences between the treatment groups were determined by One-way ANOVA with Holm-Šídák’s posthoc multiple-comparisons test. ***p <0.001, ****p < 0.0001.

To determine the potential of C5a/C5adesArg inhibition with M031 and the dual inhibition of C5 and the C3 convertase with M014, we examined the effect of these two reagents on the development of skin lesions during the disease course. Further, we evaluated the development of skin inflammation on day 12. A murine IgG1 mAb against HEL served as the isotype control Ab. Repeated injections of rabbit anti–COL7 IgG Abs into anti-HEL IgG-treated mice resulted in extensive skin lesions including erosions and blisters, sometimes covered by crusts (**Figure 1A**). The skin lesions were first visible on day 4 and steadily increased until day 12 reaching a total body surface area affected by skin lesions (ABSA) of 14.0 ± 1.5 percent (**Figure 1B**). In contrast, M031- or M014-treatment strongly attenuated the development of skin lesions. On day four, no skin lesions were visible in M014-treated mice and minor lesions in mice-treated with M031 (**Figure 1B**; left panel). Between day 6-12, the frequency of lesions was ~50% lower in M014 or M031-treated mice as compared to HEL-treated animals and reached a maximum ABSA of 6.7 ± 0.9 percent in response to M031 and 5.3 ± 1.1 percent in response to M014 treatment on day 12. In line, the individual peak values of M031 or M014-treated mice were significantly reduced as compared to HEL-treated mice (**Figure 1B**; right panel).

To evaluate one of the major hallmarks of skin inflammation, i.e. the formation of subepidermal clefts at the DEJ, we stained whole tissue sections of the ears with HE on day 12. In skin sections from anti-HEL IgG1- treated mice, we found ~ 60% of dermal-epidermal separation at the DEJ. In contrast, we observed a low frequency of only 20-25% of dermal-epidermal separation in response to M031 or M014 treatment (**Figure 1C**) which was significantly lower than that of control IgG1 Ab-treated animals.

### Prophylactic treatment with M031 or M014 reduces the infiltration of activated neutrophils at the DEJ but has no impact on IgG and C3b deposition

**Figure 2:**
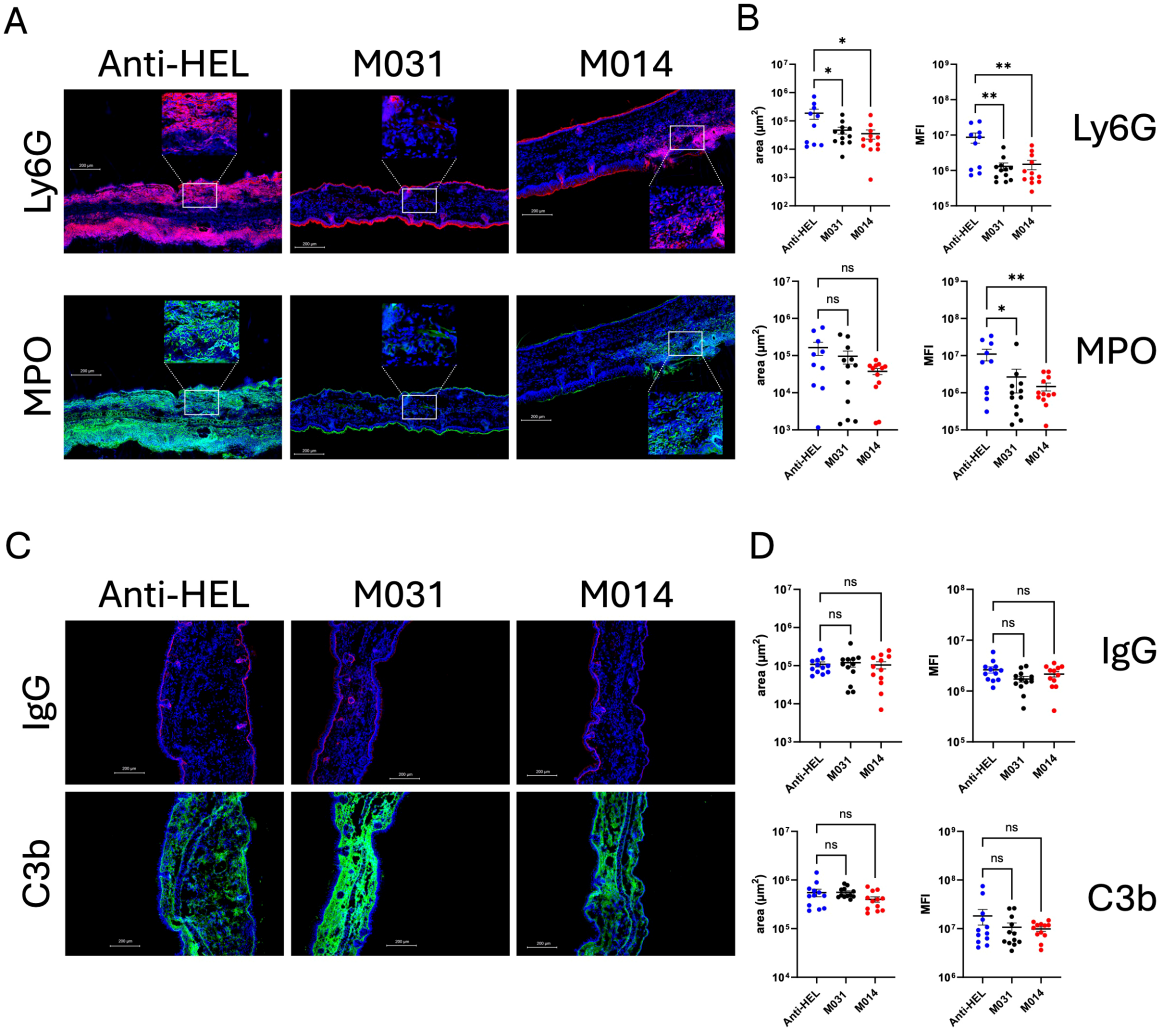
Impact of prophylactic C5a/C5adesArg- or combined AP/C5-targeting on neutrophil infiltration, IgG and C3b deposition in the skin. **(A)** Representative immunofluorescence pictures of ear skin sections from C57BL/6 mice treated with anti-HEL, M031 or M014 on day 12. Blue = DAPI; Red = Ly6G^+^ neutrophils; Green = MPO^+^ cells. The inserts show magnifications of the areas marked by the white rectangles **(B)** Quantitative evaluation of Ly6G^+^ or MPO^+^ neutrophils per μm^2^ in ear sections from mice treated with anti-HEL IgG1 (blue), M031 (black) or M014 (red). **(C)** Representative immunofluorescence pictures of ear skin sections from mice treated with Anti-HEL IgG1, M031 or M014 on day 12. Blue = DAPI; Red = IgG AAb deposition; Green = C3b deposition. **(D)** Quantitative evaluation of C3b or IgG AAb deposition per μm^2^ in ear sections from mice treated with anti-HEL IgG1 (blue), M031 (black) or M014 (red). Microscopic pictures were analyzed via Keyence analyzer software. Results in **(B, D)** were pooled from 3 independent experiments. Scatter plots show the mean ± SEM (n =10–12 mice per group). Data were analyzed using One-way ANOVA with Holm-Šídák’s posthoc multiple-comparisons test. *p < 0.05, **p < 0.01.

Recruitment of neutrophils to the skin and their subsequent activation is an important effector mechanism driving blister formation in experimental EBA (4, 18). To assess the effect of prophylactic treatment with M031 or M014 on the influx of neutrophils and expression of MPO, we determined the number Ly6G^+^ and MPO^+^ cells per μm^2^ within the whole ear skin section and the staining intensity on day 12. Immunofluorescence staining for Ly6G showed a strong influx of neutrophils (**Figure 2A**), which was significantly decreased in the M031- and M014-treated groups as compared to the anti-HEL-treated group (**Figure 2B**) whereas the number of MPO^+^ cells was only slightly decreased in response to M031 or M014 treatment.

A prerequisite for the recruitment of effector cells to the skin that drive the formation of blisters is the deposition of COL7-specific AAbs and the activation of complement at the DEJ. To assess, if prophylactic C5a/C5adesArg targeting or dual C5/AP-targeting has an impact on the deposition of IgG AAbs or C3b, we quantified the rabbit IgG and C3b deposited in the skin on day 12 by immunohistochemistry. We found IgG AAb and C3b deposition at the DEJ in all treatment groups with no differences between anti-HEL-, M031- or M014-treated mice (**Figures 2C, D**).

### Therapeutic treatment with M014 but not with M031 protects from the development of skin lesions in passive EBA

**Figure 3:**
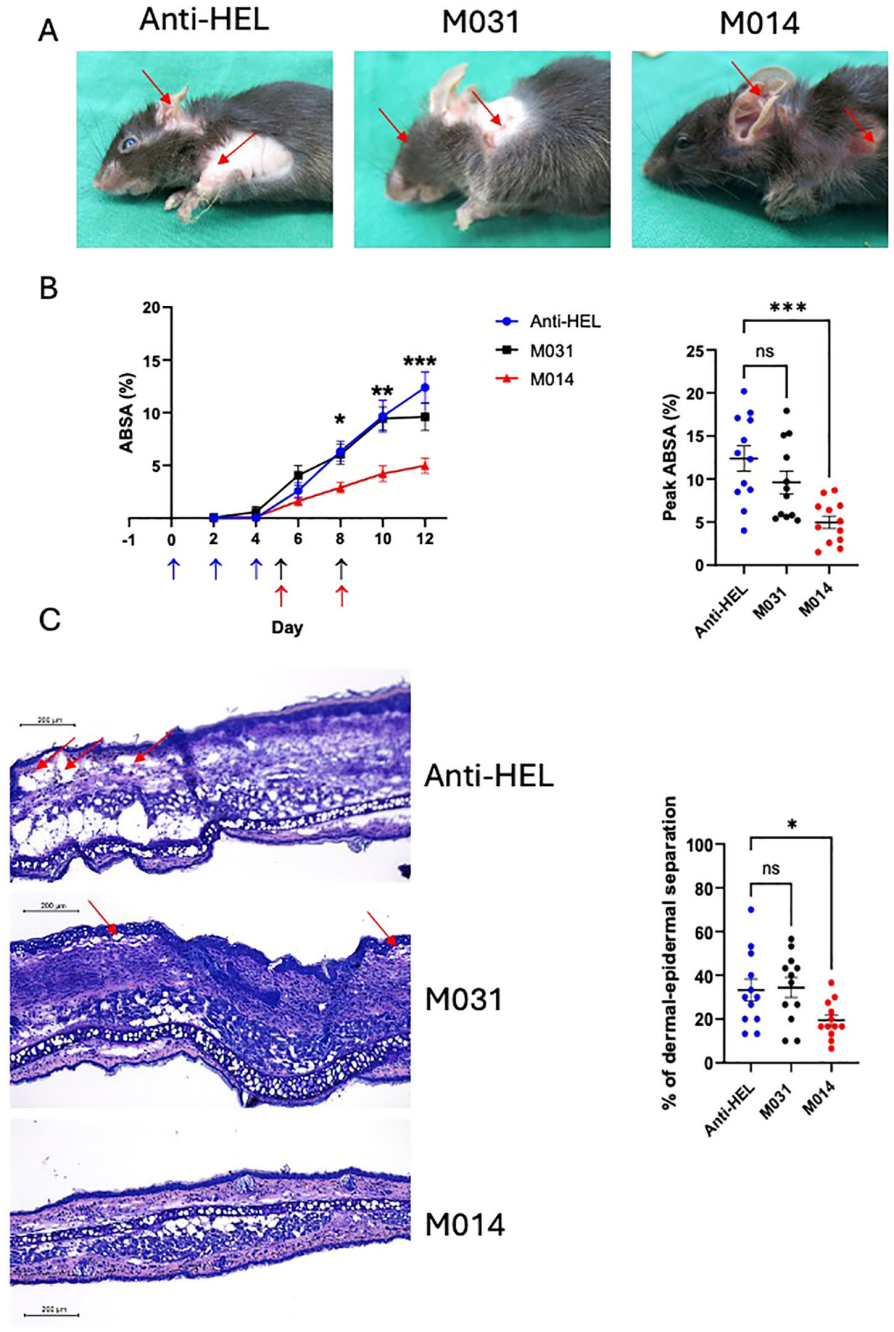
Therapeutic inhibition of AP/C5 targeting by M014 but not inhibition of C5a/C5adesArg by M031 reduces the formation of skin lesions **(A)** Representative picture of mice on day 12 after therapeutic treatment with anti-HEL IgG1, M031 or M014 on days 5 and 8. Red arrows indicate skin lesions. **(B)** (Left panel) Cumulative disease scores shown as ABSA of mice treated with anti-HEL IgG1 (blue), M031 (black) or M014 (red). Results were pooled from 3 independent experiments. Scatter plots show the mean ± SEM (*n* = 12 mice per group). Statistical differences between groups were determined by One way ANOVA with Holm-Šídák’s posthoc multiple-comparisons test. *p < 0.05, **p < 0.01. ***p < 0.001 for M014-treated compared with anti-HEL IgG1-treated mice. Blue arrows show the time point when COL7-specific IgG AAbs were injected. Black (M031) or red (M014) arrows show the time points when complement inhibitors were administered; (right panel) peak value of ABSA assessed for each mouse. ***p<0.0001. **(C)** (Left panel) Histopathologic evaluation of dermal-epidermal separation. Shown are presentative pictures of skin sections from mice treated with anti-HEL IgG1, M031 or M014 on day 12. Red arrows indicate subepidermal clefts; (right panel) percentage of dermal-epidermal separation determined individually for each mouse treated with anti-HEL IgG1 (blue), M031 (black) or M014 (red). Results were pooled from 3 independent experiments. The scatter plots show the mean ± SEM (*n* = 12 mice per group). Statistical differences between the treatment groups were determined by One-way ANOVA with Holm-Šídák’s posthoc multiple-comparisons test. *p < 0.05.

Next, we determined the therapeutic effect of treatment with M031 or M014 on the development of skin lesions. For this purpose, mice received M031 or M014 on days 5 and 8, i.e. after the appearance of first lesions on day 4. Like the prophylactic approach, we observed extensive skin lesions including erosions and blisters, sometimes covered by crusts in response to repeated rabbit anti–COL7 IgG Ab injections into anti-HEL IgG-treated mice (**Figure 3A**). First skin lesions appeared on day 4 and increased until day 12 reaching a total ABSA of 12.4 ± 1.5 percent (**Figure 3B**; left panel). In contrast to the prophylactic treatment, we found a high disease score with an ABSA of 9.6 ± 1.3 percent in mice therapeutically treated with M031 (**Figure 3B**; left panel). However, therapeutic targeting of the AP and C5 with M014 strongly attenuated the development of skin lesions. The frequency of lesions was significantly lower on days 8–12 as compared to anti-HEL-treated mice and reached a maximum ABSA of only 5.0 ± 0.7 percent on day 12 (**Figure 3B**; left panel). The individual peak value of skin lesions was significantly reduced by ~50% in the M014-treated as compared to the anti-HEL-treated group (**Figure 3B**; right panel).

When we assessed the subepidermal cleft formation at the DEJ on day 12, we found dermal-epidermal separation of DEJs in ~35% of the skin sections from anti-HEL or M031-treated mice (**Figure 3C**). In contrast, only ~20% of the DEJs from M014-treated mice showed dermal-epidermal separation, which was significantly lower than in the anti-HEL-treated group (**Figure 3C**).

### Therapeutic treatment with M031 or M014 has no impact on the infiltration of activated neutrophils, IgG and C3b deposition at the DEJ

**Figure 4:**
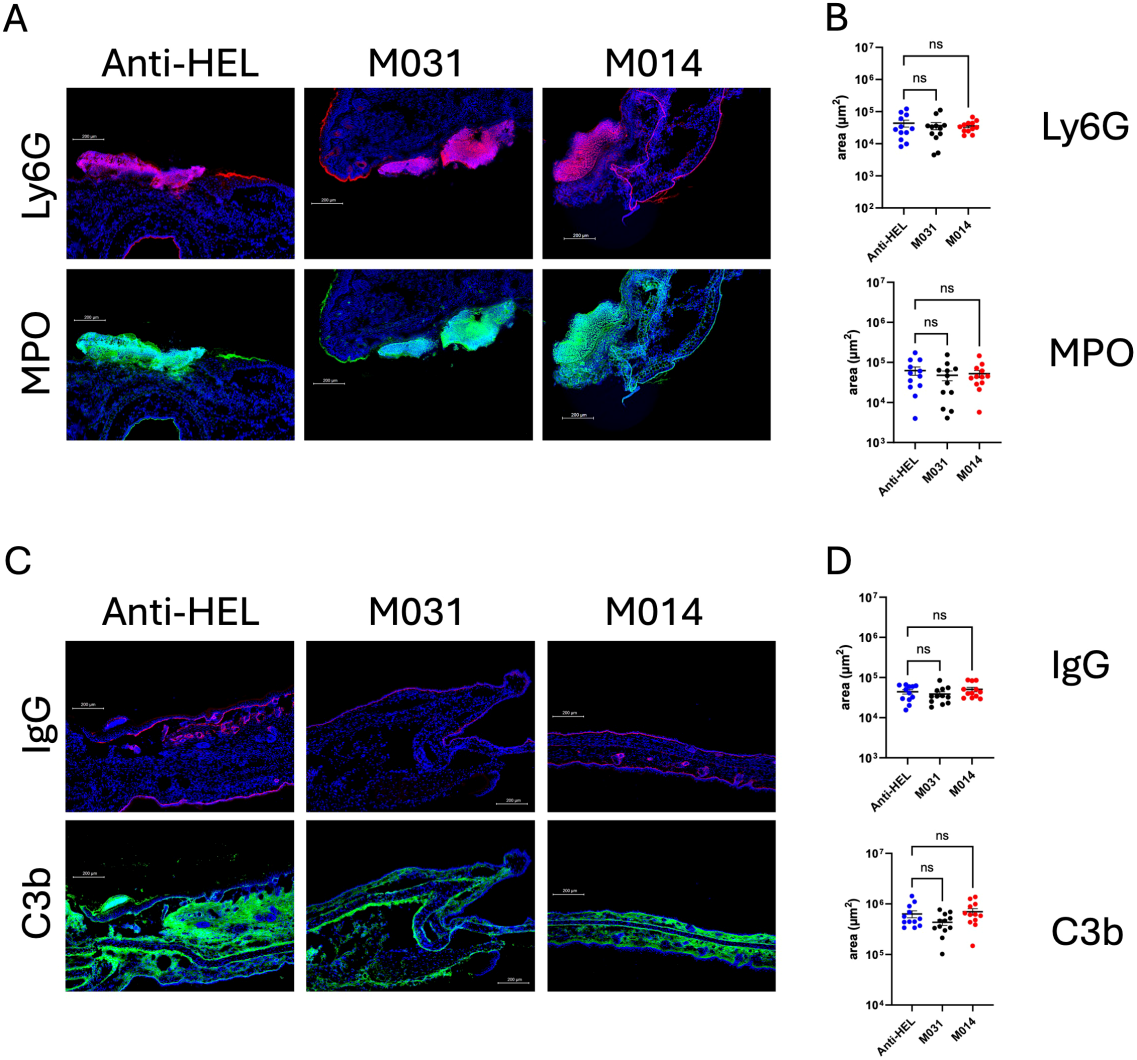
Impact of therapeutic C5a/C5adesArg- or combined AP/C5 targeting on neutrophil infiltration, IgG and C3b deposition in the skin. **(A)** Representative immunofluorescence pictures on day 12 of ear skin sections from mice treated with anti-HEL IgG1, M031 or M014. Blue = DAPI; Red = Ly6G^+^ neutrophils; Green = MPO^+^ cells. **(B)** Quantitative evaluation of Ly6G^+^ or MPO^+^ neutrophils per μm^2^ in ear sections from mice treated with anti-HEL IgG1 (blue), M031 (black) or M014 (red). **(C)** Representative immunofluorescence pictures of ear skin sections from mice treated with Anti-HEL IgG1, M031 or M014 on day 12. Blue = DAPI; Red = IgG AAb deposition; Green = C3b deposition. **(D)** Quantitative evaluation of C3b or IgG AAb deposition per μm^2^ in ear sections from mice treated with anti-HEL IgG1 (blue), M031 (black) or M014 (red). Microscopic pictures were analyzed via Keyence analyzer software. Results in B and D were pooled from 3 independent experiments. Scatter plots show the mean ± SEM (n =10–12 mice per group). Data were analyzed using One-way ANOVA with Holm-Šídák’s posthoc multiple-comparisons test. ns, not signficant.

To assess the effect of therapeutic treatment with M031 or M014 on the recruitment of neutrophils to the skin and their subsequent activation, we quantified the influx of neutrophils and expression of MPO on day 12. In contrast to the prophylactic treatment, the number of Ly6G^+^ or MPO^+^ cells in the M031- and M014-treated groups were similar to the anti-HEL group (**Figures 4A, B**). Also, we found IgG AAb and C3b deposition at the DEJ in all treatment groups with no differences between anti-HEL-, M031- or M014-treated mice (**Figures 4C, D**).

### The regulatory factor H domain contributes to early protective therapeutic effect of M014 as compared to sole anti-C5 treatment

**Figure 5:**
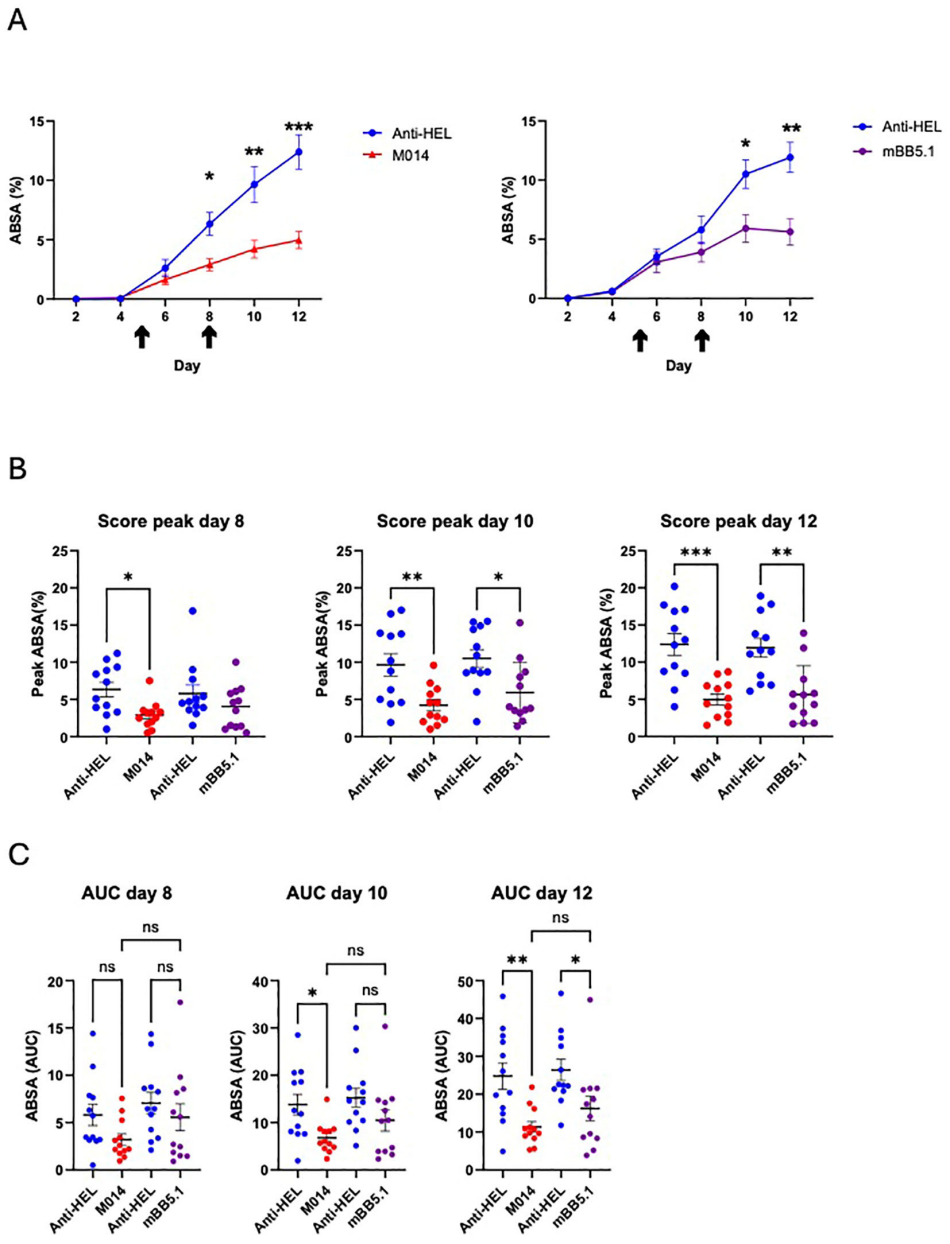
Side-by-side comparison of the therapeutic effect of M014 and mBB5.1 treatment on the development of skin lesions. **(A)** Cumulative disease scores shown as ABSA of mice treated with anti-HEL IgG1 (blue), M014 (red; left panel) or mBB51 (purple; right panel). Black arrows show the time points when the different compounds were administered (day 5 and 8). **(B)** Peak values of ABSA assessed for each mouse on days 8, 10 and 12. Scatter plots show the mean ± SEM (*n* = 12 mice per group) for the individual scoring days. *p<0.05; **p<0.01; ***p<0.001. **(C)** Area under the curve (AUC) of ABSA calculated after day 8, 10 or 12 for each mouse. Scatter plots show the mean ± SEM (*n* = 12 mice per group). *p < 0.05, **p < 0.01. ns, not significant. Data in **(B, C)** were analyzed using One way ANOVA with Holm-Šídák’s posthoc multiple-comparisons test.

In a final set of experiments, we were interested to assess the individual contribution of the regulatory factor H domain within the anti-C5 mAb M014, which controls the amplification loop of the AP pathway at the level of the C3 convertase. For this purpose, we compared the therapeutic potential of M014 directly with the parent anti-C5 mAb contained in M014. We administered both reagents on days 5 and 8 after injection of the rabbit anti-COL7 Abs. The treatment with M014 or mBB5.1 resulted in a significant reduction of skin lesions as compared to anti-HEL treatment 10 and 12 days after disease induction (**Figures 5A–C**). Importantly, evaluation of the disease peaks at the individual scoring days showed that M014 significantly reduced skin blistering already at day 8, i.e. after the 1^st^ dose of the drug, whereas mBB5.1 treatment resulted in a significant ABSA reduction only on day 10, i.e. after the 2^nd^ dose of the antibody (**Figures 5A, B**). The delayed response became also evident, when we compared the AUC until days 8, 10 and 12. While the AUC values for the period from day 0 – day 10 and day 0 – day 12 were significantly lower for M014- as compared with anti-HEL-treatment, the ABSA in response to mBB5.1 treatment was only significantly reduced during the period from day 0 – day 12 (**Figure 5C**).

## Discussion

Two types of EBA have been described, i.e. the noninflammatory mechanobullous and the inflammatory type (1). The mechanobullous form is characterized by tense blisters, skin fragility, and lesions localized to trauma-prone areas, often healing with scarring, milia, and pigmentation changes. In contrast, the inflammatory subtype resembles conditions like bullous pemphigoid or mucous membrane pemphigoid, with subepidermal separation dependent on autoantibody levels and leukocyte activation via FcγR and complement (5).

Genetic deletion or pharmacological inhibition of complement components such as C5 (9, 10), Factor B (8, 9) or C5aR1 (9, 12) markedly reduced the development of skin lesions (9, 18, 19) in the antibody-transfer model of EBA, which mimics the inflammatory EBA subtype. In contrast, C1q-, MBL-, or C6-deficiency had only a minor (8) or no effect (8, 9). These data demonstrate a marginal role of classical pathway activation by COL7-specific IgG AAbs for complement-mediated events leading to skin inflammation and the formation of lesions. In contrast, the strong protective effect of factor B or C5-deficiency point toward a key role for local complement activation by the AP and the amplification loop critical to assemble the C5 convertase, cleavage of C5 and generation of C5a. This anaphylatoxin and the activation of its two receptors, i.e. C5aR1 and C5aR2 spark the flame igniting the inflammatory events leading to the formation of skin blisters (5). Relatively little was known about the chronology of complement activation, i.e. the importance of C5a for the initiation and the subsequent boosting of inflammation, i.e. neutrophil recruitment and activation critical for dermal/epidermal separation and blister formation. We observed a strong and equally protective effect of C5a/C5adesArg targeting by M031 and of combined C5 and AP convertase targeting by M014 after prophylactic administration in the passive EBA model with significantly reduced formation of skin lesions, dermal-epidermal separation, and infiltration of neutrophils compared to animals treated with a control IgG Ab. In contrast, we observed a strong protective effect of M014 but not of M031 in the therapeutic setting, suggesting a critical role of the AP in amplifying CP activation in EBA. In support of this view, we found that mAb M014 treatment was superior to mBB5.1 treatment and protected the animals already at an earlier time point and to a greater degree.

Our findings that C5a/C5adesArg-targeting before administration of COL7-Abs but not after the development of first skin lesions protects from the influx of neutrophils, dermal/epidermal separation and skin blistering, points towards a critical role of C5a/C5aR activation as a key initiator of the local inflammatory events. C5a (20) serves as a strong chemoattractant for neutrophils, which are crucial for disease development in experimental EBA, and promotes their activation (21) including NET formation (22). Further, C5aR1 reduces the threshold for FcγR-mediated activation of innate immune cells through upregulation of activating and downregulation of inhibitory FcγRIIB (23). Thus, early complement activation by the rabbit COL7-specific IgG Abs, which bind to the DEJ within 24 hours after administration (20) can enhance neutrophil-mediated inflammation through upregulation of activating FcγRIV critical for IgG immune complex-driven neutrophil activation in this model (24). In support, we previously found in another model of IC-mediated inflammation (21) that C5aR1 and activating FcγR synergistically promote proinflammatory chemokine and cytokine release from innate immune cells. Further, combined targeting of C5 and leukotriene B4 (LTB_4_), a lipid which amplifies neutrophil recruitment, has been shown to be more efficient than simple LTB_4_ inhibition in a passive EBA model, further highlighting the important interaction between complement and effector cells as driving force in EBA (18).

**Figure 6:**
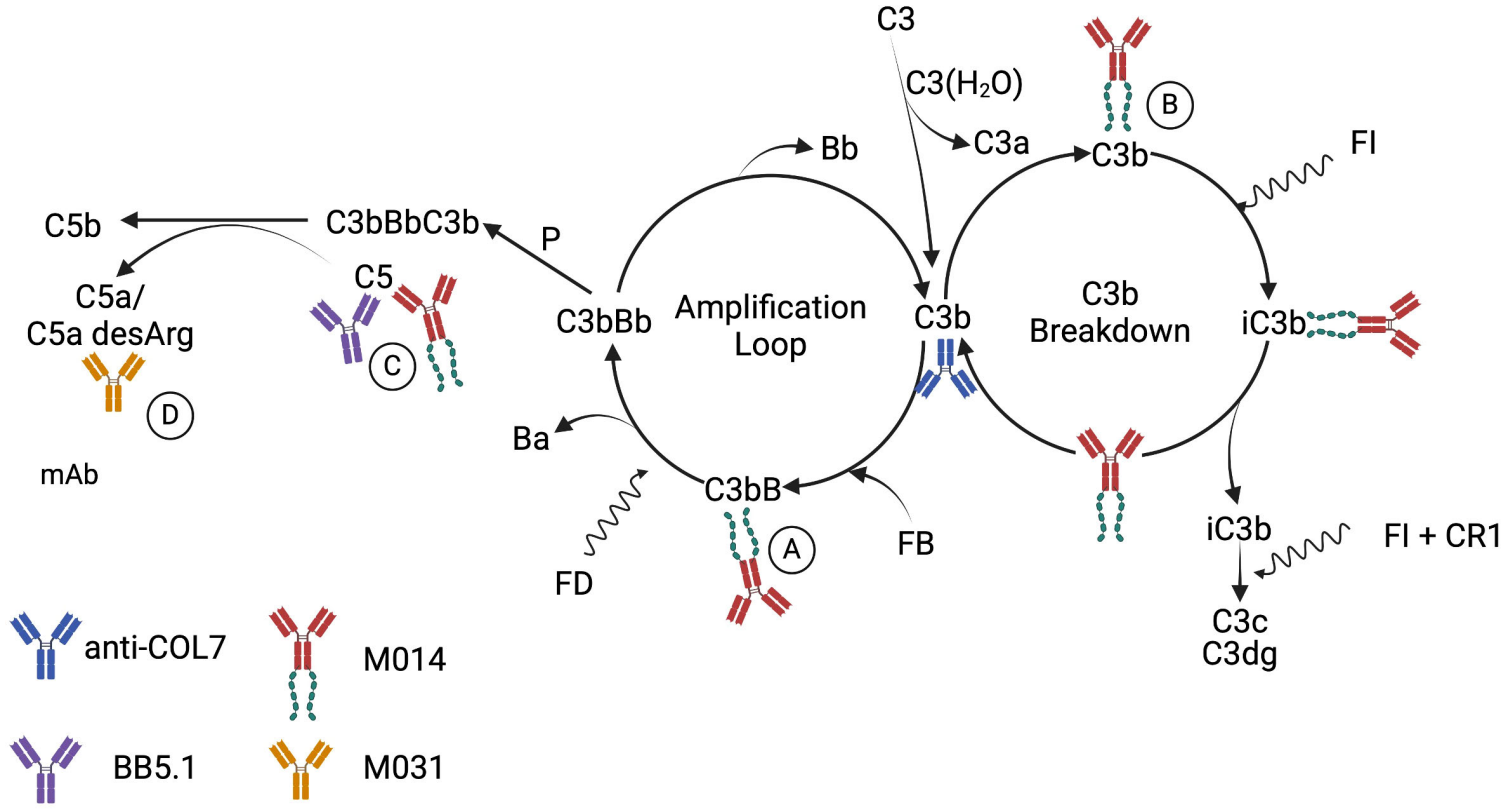
Schematic detailing the proposed mechanisms underlying the AP pathway activation by rabbit anti-COL7 AAbs and the targeting of C5, the AP and C5a/C5adesArg by mAbs mBB5.1, M014 and M031. It is well appreciated that spontaneous initiation of the AP by C3 tickover results in continuous cleavage of C3 into C3a and C3b (43, 44). Such C3b has strong affinity for IgG molecules and can form C3b2–IgG complexes (45). Also, rabbit IgG F(ab’)2 fragments can activate the AP (46). Thus, rabbit anti-COL7 IgG molecules can promote AP activation through their Fc and Fab parts. This can be amplified by C5a-dependent neutrophil activation, resulting in C3, factor B and properdin production (47). The SCR1–5 of factor H in M014 will compete with factor B for the Bb binding site on C3b leading **(A)** to the displacement of Bb and accelerated decay of the AP C3 convertase. Further, C3b-bound factor H SCR 1–5 forms a binding platform for factor I, resulting in cofactor activity and cleavage of C3b to iC3b **(B)**. Subsequently, factor I can degrade iC3b to C3c and C3dg with complement receptor 1 (CR1) as cofactor. Taken together, M014 will block the generation of C5a at several levels, i.e. upstream of C5 by: (i) the inhibition of the AP amplification loop **(A)**; and (ii) the degradation of C3b to iC3b **(B)** and directly through the inhibition of C5 cleavage by the C5 convertase of the AP or the classical pathway **(C)**. In contrast, the modified BB5.1 mAb will only prevent the cleavage of C5 by the C5 convertase **(C)**. M031 will neutralize C5a/C5adesArg **(D)** and inhibit their binding to C5aR1 or C5aR2 and the consecutive activation of neutrophils. Created in BioRender. Köhl, J. (2025) https://BioRender.com/n4q7slv.

In contrast to prophylactic treatment, only combined targeting of AP/C5, C5 alone but not of C5a/C5adesArg at the time when first lesions became evident, reduced the development of skin lesions. These findings may suggest that the local concentration of the anti-C5a mAb M031 has not been sufficient to efficiently block the large amounts of C5a/C5adesArg generated in the skin tissue from day 5 onwards and/or that other pathways are more important than C5a-driven inflammation at later time points. The result that AP/C5 or C5-blockade efficiently reduced skin lesions suggests that the C5a/C5aR pathway downstream of C5 is also important at later time points in disease development and supports the view of insufficient local C5a/C5adesArg inhibition by M031 when administered therapeutically. The more pronounced effect of combined AP/C5 targeting, which occurred earlier than C5 blockade alone, is consistent with the view that massive, AP pathway-driven generation of C5a/C5adesArg is a critical driver of neutrophil activation and subsequent skin lesion development (detailed in **Figure 6**). In support of this view, Sezin et al. (18) found high C5a serum levels of ~200 ng/ml in the passive EBA model that we used in the current study suggesting even higher local C5a concentrations in the skin tissue. Of note, non-canonical generation of C5a through cell-derived proteases seems to play a minor role as mBB5.1 only prevents the cleavage of C5 by the C5 convertase but not by other proteases. In contrast to the prophylactic treatment, we observed no protective effect on the neutrophil recruitment into the skin on day 12 in response to therapeutic administration of M014 or M031. Thus, complement activation does not seem to contribute to neutrophil recruitment but rather to neutrophil activation at later time points.

Our findings of a critical role of the C5a/C5aR pathways as drivers of skin pathology in EBA are in line with results from obtained in experimental models of other AIBDs such as BP or mucus membrane pemphigoid. In different BP models, C5/C5a have been found to attract and activate neutrophils (25) and mast cells (26). Further, prophylactic targeting of C5aR1 with the peptidic C5aR1 antagonist PMX-53 (13) or genetic deletion of C5aR1 (27) ameliorated disease development. Similarly, conjunctival and oral/pharyngeal lesions were markedly reduced in C5aR1-deficient mice in response to injection of rabbit Abs against the α3 chain of laminin 3, an immunodominant structural protein in mucus membrane pemphigoid (28). Moreover, increased systemic complement activation and expression of C5a receptors was found on immune cells detected in skin lesions from BP patients (29) confirming complement activation in human BP. Of note, expression of complement factor and complement receptors including C5aR1 has also been demonstrated on skin stromal cells such as fibroblasts and keratinocytes (30, 31), suggesting that the complement system contributes to EBA pathogenesis not only by the recruitment and activation of immune cells but fuels the immune-stromal crosstalk. Taken together, most of the available data from experimental AIBD models and from patient studies point toward a critical role for C5/C5a/C5aR axes activation in disease pathogenesis.

The treatment regimen for AIBDs has not much changed during the past decades and includes topic or systemic corticosteroids alone or in combination with dapsone, tetracycline, cyclophosphamide or azathioprine (32). In the aged population of AIBD patients, such treatment is frequently associated with severe side effects that overweigh the benefits. Thus, several novel treatment approaches have been proposed, several of which are currently evaluated in clinical trials (33). In a phase I study, inhibition of the CP at the level of C1s partially or completely abrogated C3c deposition at the DEJ, suggesting that CP activation in BP contributes to complement activation (34). This finding may explain why combined AP/C5 inhibition by M014 did not reduce C3b deposition in this study, i.e. the lack of CP inhibition by this approach resulting in C4b2a, which can cleave C3 into C3a and C3b. In two proof-of-concept studies, C5aR1- or combined C5/LTB4 targeting has been assessed as an additional therapy to corticosteroids with inconclusive results. While C5aR1-targeting showed no efficacy (35), C5/LTB4 inhibition resulted in >80% reduction in disease activity in 3 or 40% in another 3 out of 9 patients (36). Clearly, well-designed placebo-controlled studies are warranted to fully explore the potential of complement targeting in AIBD. For this purpose, several approved complement drugs are available that allow targeting the complement cascade at the level of the CP, the AP, C5, C5a or the C5aR1 (reviewed in (37).

Taken together our data demonstrate that combined therapeutic targeting of the AP and C5 effectively inhibits the progression of skin lesions in experimental EBA during the effector phase. The urgent need to improve the therapeutic options in AIBD warrants the clinical evaluation of combined AP and C5 targeting. Such treatment must take in to account the important role of the AP and the terminal pathway in host defense. Over the past 18 years, long-term targeting of C5 in patients with paroxysmal nocturnal hemoglobinuria (PNH) has been shown to be highly beneficial with a remarkably safety profile. However, blocking C5 exposes patients to a high risk of infection with encapsulated bacteria, i.e. *Neisseria meningitidis*, due to impaired MAC generation, which can be largely mitigated by vaccination and antibiotic prophylaxis (38). At present there are no clinical data available on adverse effects in response to targeting the AP with factor H SCR1-5. However, C3 blockade with the peptide inhibitor pegcetacoplan requires vaccination against *Streptococcus pneumoniae, Neisseria meningitidis* and *Haemophilus influenzae* and prophylactic antibiotic treatment. The available data suggest that this regimen keeps the risk of infection very low (39).

Considering emerging data showing a critical role for C3aR and C5aR1 in the regulation of the B cell response in germinal centers (40) including T follicular helper cell differentiation (41) and class-switch recombination (42), AP and C5 targeting may also suppress the generation of COL7-specific AAbs, which should be assessed experimentally in the immunization-induced model of EBA (7) in the future.

## Data Availability

The original contributions presented in the study are included in the article/supplementary material. Further inquiries can be directed to the corresponding author.
